# Green Synthesis of Iron-Doped Cobalt Oxide Nanoparticles from Palm Kernel Oil via Co-Precipitation and Structural Characterization

**DOI:** 10.3390/nano11112833

**Published:** 2021-10-25

**Authors:** Cedrik Ngnintedem Yonti, Patrice Kenfack Tsobnang, Roussin Lontio Fomekong, Francois Devred, Eric Mignolet, Yvan Larondelle, Sophie Hermans, Arnaud Delcorte, John Lambi Ngolui

**Affiliations:** 1 Inorganic Chemistry Department, University of Yaoundé I, Yaoundé 812, Cameroon; cedrik.ngnintedem@uclouvain.be; 2 Institute of Condensed Matter and Nanosciences, Catholic University of Louvain, Croix du Sud, B-1348 Louvain-la-Neuve, Belgium; francois.devred@uclouvain.be (F.D.); sophie.hermans@uclouvain.be (S.H.); arnaud.delcorte@uclouvain.be (A.D.); 3 Chemistry Department, Faculty of Science, University of Dschang, Dschang 67, Cameroon; pakenfack@gmail.com; 4 Chemistry Department, Higher Teacher Training College, University of Yaoundé I, Yaoundé 47, Cameroon; jngolui@gmail.com; 5 Louvain Institute of Biomolecular Science and Technology, Catholic University of Louvain, Croix du Sud, B-1348 Louvain-la-Neuve, Belgium; eric.mignolet@uclouvain.be (E.M.); yvan.larondelle@uclouvain.be (Y.L.)

**Keywords:** green synthesis, palm kernel oil, carboxylate fatty acids, co-precipitation method, Fe-doped Co_3_O_4_

## Abstract

In this study, a bio-derived precipitating agent/ligand, palm kernel oil, has been used as an alternative route for the green synthesis of nanoparticles of Fe-doped Co_3_O_4_ via the co-precipitation reaction. The palm oil was extracted from dried palm kernel seeds by crushing, squeezing and filtration. The reaction of the palm kernel oil with potassium hydroxide, under reflux, yielded a solution containing a mixture of potassium carboxylate and excess hydroxide ions, irrespective of the length of saponification. The as-obtained solution reacts with an aqueous solution containing iron and cobalt ions to yield the desired metallo-organic precursor, iron cobalt carboxylate. Characterization of the precursors by IR and gas chromatography (GC) attests to the presence of carboxylate fatty acids in good agreement with the proportion contained in the oil, and ICP confirms that the metallic ratios are in the proportion used during the synthesis. Analysis of the products thermally decomposed between 400 °C and 600 °C by XRD, EDX, TEM and ToF-SIMS, established that cobalt iron oxide nanoparticles (Co_(1−x)_Fe_x_)_3_O_4_ were obtained for x ≤ 0.2 and a nanocomposite material (Co_(1−x)_Fe_x_)_3_O_4_/Fe_3_O_4_ for x ≥ 0.2, with sizes between 22 and 9 nm. ToF-SIMS and XRD provided direct evidence of the progressive substitution of cobalt by iron in the Co_3_O_4_ crystal structure for x ≤ 0.2.

## 1. Introduction

In the last three decades, much research has been devoted to the synthesis and characterization of materials at the nano scale. Nanomaterials exhibit unique properties with respect to their bulk counterparts mainly because of size-dependent effects [1]. As the size of a system decreases, the surface area increases, changing mechanical, thermal, optical and catalytic properties significantly [2]. Transition metal oxide nanoparticles have attracted considerable attention and cobalt oxide, in particular, exhibits specific chemical and thermal stability [3]. It is well known as a photocatalyst [4], a catalyst in N_2_O decomposition [5], a highly selective CO sensor [6], a high temperature solar selective absorber [7], an electrode material for thin film supercapacitor [8], a magnetic material [9] and it is used in electrochromic devices [10], because its optical properties changes under an external electrical stimulus.

Co_3_O_4_ is a black antiferromagnetic p-type semiconductor material, which crystallizes in a normal spinel structure with the chemical formula AB_2_O_4_, based on a cubic close-packing structure of oxide ions, with Co^2+^ ions occupying eight tetrahedral A-sites and Co^3+^ ions occupying 16 octahedral B-sites. Co_3_O_4_ nanoparticles have been extensively studied [11,12,13,14]. In particular, the modification of their properties by progressively substituting the Co metal ions in their structure with other metal ions (Mg and Ni [14], Fe [5,15,16], Mn [17,18], Cd [19], Cu [20], Pd [21], Mo [22]), thus, forming a mixed cobalt oxide, is rather well established. Thus, the synthesis of cobalt oxide and mixed cobalt oxides using various routes has been reported, including the facile solvothermal route [23], chemical spray pyrolysis [7], chemical vapor deposition [10], sol-gel [24], hydrothermal [25], simple combustion [26] and co-precipitation methods [5]. Co-precipitation, in particular, is a simple, low-cost method that is used for the preparation of simple metal oxides and hetero-atom metal oxides. The main advantages of this synthesis method are that it allows the control of the stoichiometry of the final product and that it does not require a sophisticated high vacuum or high temperature system. For example, using this method, mixed cobalt iron oxide nanoparticles could be obtained by thermally decomposing a precursor pre-synthesized via the reaction between a cobalt iron salt solution and an O-donor ligand.

Many O-donor ligands (carboxylates) have been used for the synthesis of simple and mixed metal oxides in the last few decades like oxalates [15,17], malonates [27,28], succinates [28,29], acetylacetonates [30,31] and octanoates [32]. Most of the carboxylate ligands used for that purpose are generally synthetic whereas some are readily available in our environment (mostly in plants [33]). They can be used as a green and renewable source of ligand for the synthesis of the precursor. These green sources include, amongst others, citric acid, tartaric acid, oxalic acid and the linear long-carbon chain fatty acids. The linear long-carbon chain fatty acids are the major component of every vegetal oil, especially palm kernel oil. They are present in vegetable oils in the mono-, di- or tri-ester form from which the corresponding acid can be easily released by a simple saponification reaction. Therefore, the possibility of using this natural, readily available, renewable and sustainable source of carboxylate ligands for the synthesis of simple and hetero-metal oxide nanoparticles of cobalt and iron is explored in this study. It could also open up the possibility of high exploitation of non-edible oil. Many of such green syntheses have been reported recently for the synthesis of cobalt oxide nanoparticles [34,35,36,37,38,39,40,41,42], even though palm kernel oil was not used as in the present study.

Palm kernel oil is extracted from the palm kernel/nuts of the palm oil tree, *Alaeis guinensis*, and contains about 82% saturated fatty acid. In Cameroon, it is mostly used for the fabrication of soap and in the skin protection while in the USA, Europe and Malaysia, hydrogenation and fractionation products of palm kernel oil are used for chocolate-type couvertures, biscuit cream fillings, sugar confectionery, coffee creamers, imitation creams, to replace butterfat in filled milk [43,44,45].

In this paper, we report, for the first time and to the best of our knowledge, the use of a carboxylate ligand extracted from palm kernel oil, a Cameroonian local oil product, as the precipitating agent for synthesis of mixed cobalt-iron oxide, (Co_(1−x)_Fe_x_)_3_O_4_ (x ≤ 20) and nanocomposite materials, (Co_(1−x)_Fe_x_)_3_O_4_/Fe_3_O_4_ (x > 20) by thermally decomposing the precursor (hetero-metal carboxylate) pre-synthesized via a simple co-precipitation reaction The nature of the precursor was partially elucidated using Fourier transform infrared (FTIR) spectroscopy, thermogravimetric analysis (TGA), gas chromatography (GC) and inductively coupled plasma-atomic emission spectroscopy (ICP-AES). The structure and stoichiometry of the hetero-atom metal oxide nanoparticles obtained were determined by XRD, FTIR, EDX-SEM, ToF-SIMS, XPS and TEM.

## 2. Materials and Methods

### 2.1. Materials

Palm kernel seeds were purchased in the local market. Cobalt(II) chloride hexahydrate (CoCl_2_·6H_2_O, 98%, Sigma Aldrich, St. Louis, MO, USA), Cobalt(II) nitrate hexahydrate (Co(NO_3_)_2_·6H_2_O, ≥99.0%, Sigma Aldrich, Darmstadt, Germany), Iron(III) nitrate nonahydrate (Fe(NO_3_)_3_·9H_2_O, Sigma Aldrich, St. Louis, MO, USA) potassium hydroxide (KOH, ≥85% Carl Roth, Karlsruhe, Germany), sulfuric acid (95%, VWR, Fontenay sous bois, France), nitric acid (65%, VWR, Darnstadt, Germany) and hexane (97%, VWR, Gliwice, Poland) were used as received, without further purification.

### 2.2. Methods

(Co_(1−x)_Fe_x_)_3_O_4_ and (Co_(1−x)_Fe_x_)_3_O_4_/Fe_3_O_4_ nanoparticles were obtained via five experimental steps: 1. Extraction of palm kernel oil from palm kernel seeds; 2. Synthesis of the carboxylate (using the saponification reaction); 3. Titration of the carboxylate; 4. Synthesis of cobalt iron carboxylate precursors and finally 5. Thermal decomposition of the precursors.

#### 2.2.1. Extraction of Palm Kernel Oil

The extraction of the palm kernel oil from palm kernel seeds was carried out using classical traditional methods in the western region of Cameroon. The palm kernel seeds were spread out on a metal plate with small holes connected to a terracotta oven. After drying, the palm kernel seeds were then poured into an electric grinder which separates the hot black palm kernel oil from the pulp of the palm kernel shells. Filtration then yields the pure yellow palm kernel oil.

#### 2.2.2. Carboxylate Synthesis

The carboxylate ligands were generated via a saponification reaction between the palm kernel oil (9.98 g) and 30 mL of aqueous KOH (1.375 mol/L), at 96 °C, under reflux, varying the time of the saponification reaction (2 h, 3 h, 6 h, 7 h, 8 h). The same reaction was carried out by doubling the oil mass for 2 h of reaction time. Each obtained solution was transferred to a decanted funnel containing 10 mL of hexane. The mixture was homogenized and left to stand for 24 h. It forms two phases, an aqueous phase containing carboxylates, and a non-aqueous phase.

#### 2.2.3. Titration of Carboxylate Solution

The amount of carboxylate in the aqueous phase is determined by pH-metric titration. The pH-meter was calibrated by two buffer solution (pH = 7 and 10). 5 mL of the aqueous phase are homogenized in 15 mL of distilled water and titrated with sulfuric acid (0.25 M). From the titration curves, we determined the concentration of the obtained carboxylates, the remaining hydroxide ions, and deduced the amount of metal ions likely to react.

#### 2.2.4. Synthesis of the Cobalt Iron Carboxylate Precursors

The titration curves allowed us to adopt two synthesis routes: first synthesis route and second synthesis route, both depending on the nature of the carboxylate solution obtained in Section 2.2.2 above. The same amount of the carboxylate solution; for each synthesis route, was used to synthesize cobalt iron carboxylate precursors The first synthesis route used the carboxylate solution as obtained, resulting from the saponification reaction between 9.98 g of palm kernel oil and 30 mL of KOH solution (1.375 mol/L) during 2 h. For example, in order to obtain a precursor containing 90% of cobalt and 10% of iron (mole/mole), an aqueous solution of 0.516 g of CoCl_2_·6H_2_O and 0.097 g of Fe (NO_3_)_3_·9H_2_O was added dropwise in a beaker containing 5 mL of potassium carboxylate solution. The precipitate formed was rinsed with distilled water and dried. The second synthesis route used the carboxylate solution resulting from the saponification reaction between the double mass of oil (19.8 g) and 30 mL of KOH solution (1.375 mol/L). All the excess hydroxide ions were neutralized by nitric acid prior to the addition of the metallic solution. For example, for the synthesis of the precursor containing the same proportion of cobalt and iron as above, 2.5 mL of nitric acid (0.25 M) were added dropwise using a burette to 3.8 mL solution of potassium carboxylate. To this solution, an aqueous solution of 0.218 g of Co(NO_3_)_2_·6H_2_O and 0.0337 g of Fe(NO_3_)_3_·9H_2_O was added dropwise. The precipitate thus obtained was then rinsed with distilled water and dried.

Samples are designated by the letter S (first synthesis route), S’ (second synthesis route) and a number which represents the expected percentage of iron in the compound. For example, sample S10/S’10 is the one that contains 10% of iron and 90% of cobalt. The carboxylate ligand is represented by RCOO. The amounts of metallic reagents used during the synthesis are presented in Table 1.

#### 2.2.5. Thermal Decomposition of the Precursors

The cobalt iron carboxylates obtained were thermally decomposed in a ceramic combustion boat holder between 400 °C and 600 °C in a thermal oven for one hour at a heating rate of 10 °C/min under air atmosphere. The thermal decomposition process/temperature was followed/determined by thermogravimetry.

#### 2.2.6. Characterization of the Precursors and Final Products

Metallic elemental analyses of the precursors were performed via inductively coupled plasma-atomic emission spectroscopy (ICP-AES) using the Thermo scientific ICAP 6500 Duo (Watham, MA, USA). ~40 mg of sample were digested in 4 mL of mixed acid (3 mL of concentrated nitric acid and 1 mL of concentrated hydrochloric acid) and the mixture diluted with 500 mL distilled water. Measurements were carried out on this final solution.

The functional groups in the precursors and the calcined products were determined using the Nicolet Nexus 870 Fourier transform infrared (FTIR) spectrometer (Madison, WI, USA) and the Thermo Scientific Nicolet iN10 infrared microscope (Waltham, MA, USA) in transmission mode. All samples were prepared by the method of KBr pellets except precursors obtained by the second synthesis route which were simply squeezed onto a transparent KRS-5 crystal. The spectra were recorded with 64 scans at 4 cm^−1^ resolution for the Nicolet Nexus 870 spectrometer and with 64 scans at 16 cm^−1^ for the Scientific Nicolet iN10 infrared microscope, all in the 4000–500 cm^−1^ range, in transmission mode.

The content of the carboxylates present in the precursors was determined by gas chromatography and then compared with those present in the oil. Carboxylates were slowly released from the metallo-organic precursors by their reaction with 50 mL of sulfuric acid 0.25 M at about 70 °C under magnetic stirring. This formed an oily layer of carboxylic fatty acid, which coagulated after cooling. It was washed, filtered and, as for palm kernel oil, esterified by the method described by Folch et al. [46]. This method involves a saponification with 0.1 M KOH/MeOH at 70 °C for 1 h, followed by esterification using a 1.2 M HCl/MeOH solution at 70 °C for 20 min. An HPLC grade hexane-water solvent (95:5) was used to extract the methylated fatty acids. They were finally analyzed by GC trace gas chromatography (Thermoquest; Milan, Italy) equipped with a flame ionisation detector. For the chromatographic separation, a restek RT2560 capillary column (0.25 mm in diameter, 100 m in length) coated with a polar stationary phase of 0.2 µm thickness (Bellefonte, PA, USA) was used. The carrier gas, hydrogen, was maintained at a constant pressure of 200 KPa. The column of temperature was programmed as follows: 80 °C for 0 min; 80–175 °C for 3.8 min (25 °C/min); 175 °C for 30 min; 175–205 °C for 3 min (10 °C/min); 205 °C for 4 min; 205–225 °C for 2 min (10 °C/min); 225 °C for 20 min. and 225–80 °C for 7.25 min (20 °C/min). The detector temperature was set at 280 °C. Hydrogen and air flow rates for the detector were maintained throughout all runs at 35 and 350 mL/min, respectively. A calibration mixture of fatty acids standards was processed in parallel. The data were analyzed by a chromquest 3.0 software (Thermo Fisher, Waltham, MA, USA).

Thermal behavior of the precursor was studied by thermogravimetric analysis (TGA) on a METTLER TOLEDO Thermal Analyzer (Columbus, OH, USA) in air at a flow rate of 100 mL.min^−1^, a heating rate of 10 °C min^−1^ and a temperature range of 25–600 °C/900 °C.

XRD measurements of the calcined precursors were performed using a Bruker D8 advanced diffractometer (Bruker, karlshube, Germany) equipped with a linkeye XE-T detector and Cu source. The two-theta range from 5 to 80° was scanned with an increment of 0.015° and an integration time of 0.15 s using a Bragg Brentano geometry. For the experiment, the decomposition product was spread out on the silicon plate in such a manner as to avoid preferred orientations. The Bruker software DIFFRAC.EVA (version V4.2, Karlsruhe, Germany) was used for data processing using either the COD or PDF 2 database for phase identification.

Raman spectroscopy was carried out using a confocal Microscope DXR Raman ThermoScientifc inc. model (Madison, WI, USA) equipped with a diode light (785 nm). The resolution was set to 4 cm^−1^. The number of scans was 10 and the time of accumulation was 10 s per scan. The laser power was set to 10 mW and the 50× objective was used.

The surface chemical composition of the decomposition product was determined by X-ray photoelectron spectroscopy (XPS) using a SSX 100/206 photoelectron spectrometer from Surface Science Instruments (Mountain view, CA, USA) equipped with a monochromatized micro focused Al X-ray source (powered at 20 mA and 10 kV).

The morphologies of the sample were investigated by transmission electron microscopy using the TEM Leo922 (Zeiss, Germany) with an accelerating voltage of 120 kV. TEM samples were prepared by dropping a sonicated water dispersion suspension of the powder samples on a carbon-coated copper grid.

Chemical characterisations of the samples were carried out using a TOF.SIMS^5^ instrument (IONTOF GmbH, Münster, Germany). A pulsed Bi_5_^+^ metal ion source was used to produce a primary beam with an acceleration voltage of 30 kV to bombard powder samples pressed onto the adhesive part of Post-it^®^ papers. An AC target current of 0.08 pA with a bunched pulse width lower than 1 ns was used. Both positive and negative secondary ion species were analysed. For spectra acquisition, a raster of 128 × 128 data points over an area of 250 × 250 µm^2^ was used. The total primary ion beam dose for each analysed area was always kept below 5 × 10^10^ ions.cm^−2^, ensuring static conditions. Lateral resolution of ~3 µm and mass resolution m/Δm > 5000 at 29 *m*/*z* were maintained for positive and negative spectra acquisition. Charge compensation was achieved by interlaced electron flood gun (E_k_ = 20 eV). All data analyses were carried out using the software supplied by the instrument manufacturer, SurfaceLab (version 6.8; Münster, Germany).

## 3. Results and Discussion

### 3.1. Titration Curves of the Carboxylates Solutions

**Figure 1:**
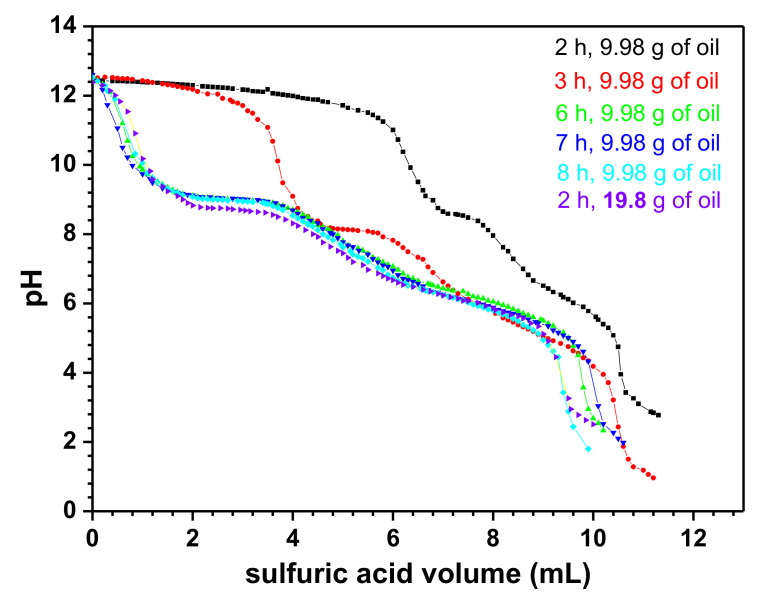
The pH-metric titration curve between obtained carboxylate solution and sulfuric acid solution.

Figure 1 presents the titration curves of the carboxylate solutions obtained.

The average concentration of all the carboxylate solutions is 1.01 mol/L. All curves look like a titration of a strong acid with a solution containing both strong and weak base. This means that, in addition of the carboxylate formed, hydroxide ions (strong base) remain in the solutions obtained. The quantity of remaining hydroxide ions decreases with the time of the saponification reaction from 2 h until 6 h and then, remains constant. However, the same quantity of hydroxide ions is obtained for only 2 h when the initial quantity of oil is doubled. Thus, two different synthesis routes have been adopted: Using the carboxylate solution as obtained for 2 h/9.98 g of oil of the precursor (first synthesis route), and using the carboxylate obtained for 2 h/19.8 g of oil prior to the neutralization of all the excess hydroxide ion (second synthesis route).

### 3.2. ICP-AES Analysis of the Precursors

The ratios between metals contained in the precursors were determined using ICP-AES. The results are presented in the Table 2 and are compared with those calculated from the concentrations of reagents.

The results reveal that the ratios of metallic ions present in the precursor correspond to the expected value which confirms that the synthetic method adopted was good.

### 3.3. Fourier Transform Infrared (FTIR) Spectral Characterization of the Precursor

**Figure 2:**
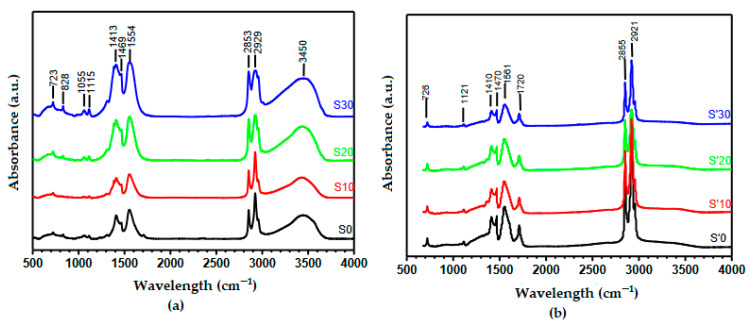
Fourier transform infrared (FTIR) spectra of samples (**a**) S0, S10, S20, S30 and (**b**) S’0, S’10, S’20, S’30.

Figure 2 shows IR spectra of the obtained precursors. To this effect, samples S0, S10, S20 and S30 were selected as being more representative of the eight samples for the first synthesis route while samples S’0, S’10, S’20, S’30 were chosen for the second synthesis method.

Figure 2 shows that in all the samples, the peaks characteristic of the symmetric and asymmetric stretching vibrations of carboxylic group OCO are observed, respectively, at 1410–1413 cm^−1^ and 1554–1561 cm^−1^. This attests the presence of carboxylate groups in the samples. The peaks at 1297 and 1469 cm^−1^ are being assigned to the bending vibrations of the C–H while that at 1115–1120 cm^−1^ is being attributed to C–O stretching and that at 723–726 cm^−1^ to the bending deformation of OCO. The intense peaks at 2853 cm^−1^ and 2929 cm^−1^ are typical of the aliphatic symmetric and asymmetric stretches, respectively, of the –CH group. It should be noted that the peak at 1720 cm^−1^ characteristic of the C=O double bond of the carboxylate group present in the S’ samples, is practically absent in the S samples. Since for all the S samples obtained by the 1st synthesis route there is no residual carboxylate acid present, it implies that the entire carboxylate group is engaged in the formation of the coordination compound. Kamta et al., working with the octanoate ligand (a C-8 straight-chain carboxylate) and employing the same synthesis method, obtained similar results [32]. The prominent –OH peak observed at 3450 cm^−1^ for S samples (Figure 2a) are absent in the S’ samples (Figure 2b). This is ample proof that all the excess OH^−^ ions were completely neutralized in the S’ samples prior to the reaction with the metal salt solutions.

### 3.4. Gas Chromatography (GC) Analysis

Table 3 compares the carboxylate contents (%) in the precursors and in the palm kernel oil.

These results in Table 2 show that the palm kernel oil used in this work is saturated at 86.25% with 47.16% of lauric acid. This is in agreement with the literature which estimates the average percentage of saturated fatty acid at about 82% with 48% of lauric acid [45,47]. The high proportion of saturated fatty acid found in the precursors (88.33% of saturated acid with 52.75% of lauric acid) indicates that the carboxylates present in the oil are almost completely released in solution irrespective of their molar masses. From Table 3, it is evident that the relative proportions of the carboxylates present in the palm kernel oil and in the metallo-organic precursors are comparable. The table also shows that the principal fatty acids in the palm kernel oil and the precursors are, respectively lauric acid, myristic acid, oleic acid and palmitic acid, caprilic, capric, linoleic and stearic.

### 3.5. Thermogravimetry Analysis of the Precursors

**Figure 3:**
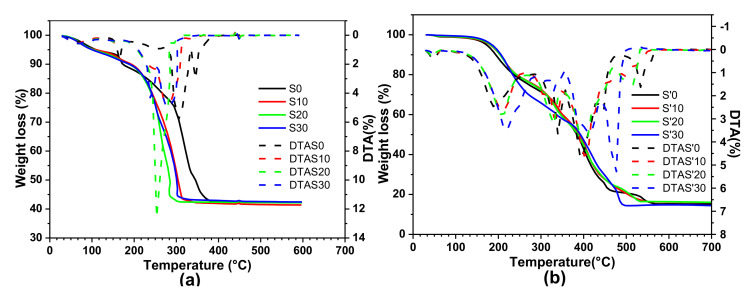
Thermogravimetric analysis (TGA) and differential thermal analysis (DTA) curves for samples (**a**) S0, S10, S20, S30; (**b**) S’0, S’10, S’20, S’30.

Figure 3a,b give the comparative thermogravimetric and differential thermal analysis (DTA) curves of S samples (S0, S10, S20, S30) and S’ samples (S’0, S’10, S’20, S’30), respectively. The weight loss peaks have been determined by a graph of the weight difference for consecutive rows against the temperature (called DTA curves). The thermogravimetric (thermograms) and DTA curves, of all the samples synthesized with the same synthesis route have similar thermal behavior. However, it should be noted that for samples obtained by the first synthesis method, those in which cobalt is substituted by iron (S10, S20, S30) decompose at a lower temperature.

DTA curves of S samples S0, S10, S20, S30 show that their decompositions follow three main steps. Taking the DTA curve for the S0 as an illustration, the maximum weight losses occurs around 165, 305 and 345 °C. The weak weight loss (~5.9%) observed between 70 °C and 170 °C is attributed to 0.75 molecules of water of crystallization. A major peak (48.18%) occurring between 170 °C and 370 °C is being attributed to the loss of the organic part of the precursor. All the decomposition product residues correspond to a total weight loss of ~61% and no further weight loss is observed beyond 370 °C. Therefore, this explains why 400 °C was adopted as the thermal decomposition temperature for all the S samples. On the other hand, DTA curves for the S’ samples (S’0, S’10, S’20, S’30) show five main steps, generally with some overlapping. For example, for the S’0 sample, the weight loss peaks occur around 190, 340, 400, 450 and 553 °C. They all completely decompose around 570 °C with a total weight loss of 85.3%, the reason why 600 °C was chosen as the decomposition temperature for all the S’ samples. Comparing the total weight loss of ~61% for the S samples (those with OH^−^ ions present) to that of 85.3% for the S’ samples (those with the carboxylate group linked directly to the metal), it is evident that the S’ samples yield the desired results given that their theoretical total weight loss is 84%. This assertion is confirmed by the absence of an –OH peak at 3450 cm^−1^ in the IR spectra for the S’ samples (see the –OH peak observed for the S samples). According to previous results published by Kamta et al. [29], the formula proposed for the compounds synthesized with the first synthesis route (S samples, in our case) is Co(RCOO)_0.7_(OH)_1.3_·0.75H_2_O while that for samples obtained via the second synthesis route (S’ samples in our case) is Co(RCOO)_2_ (where RCOO corresponds globally to the carboxylate groups in Table 2).

### 3.6. FTIR Spectral Characterization of the Decomposition Products

**Figure 4:**
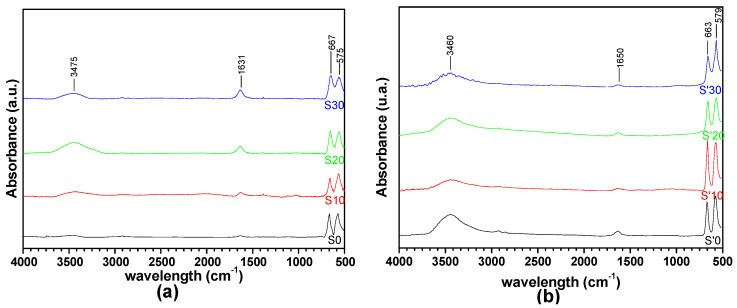
Infrared (IR) spectra of the decomposition products obtained from (**a**) S samples (S0, S10, S20, S30) calcined at 400 °C and (**b**) S’ samples (S’0, S’10, S’20, S’30) calcined at 600 °C.

Figure 4 represents the IR spectra of the products obtained from the thermal decomposition at 400 °C and 600 °C, respectively, of the precursors of the S samples (S0, S10, S20, S30) and the S’ samples (S’0, S’10, S’20, S’30). As the spectra indicate, the bands often due to the carboxylate (OCO) and aliphatic carbon-hydrogen (–CH) groups are absent. On the other hand, new peaks appear at 578 cm^−1^ and 670 cm^−1^ which are attributed, respectively to the stretching vibrations of the Co^3+^-O-Co^3+^ and Co^3+^-O-Co^2+^ moieties, indicative of the formation of a Co_3_O_4_ spinel structure [9,21]. Peaks associated with the stretching vibration of the water/OH^−^ group at 3450 cm^−1^ and the bending vibration of water at 1615 cm^−1^ are also observed. Even though it is not usual to find peaks due to water after calcination at 400 °C/600 °C, Makhlouf et al. have observed similar –OH in the spectra of Co_3_O_4_ obtained after the calcination of cobalt oxalate precursors at 773 K (500 °C) [9]. Lontio et al. also found water in the Ni_1−x_Zn_x_O and Ni_1−x_Zn_x_O/ZnO spectra obtained after calcination of the nickel zinc malonate precursor at 500 °C [27]. This tendency for metal oxide to bind hydroxide group on their surface has equally been reported by Gengnan Li et al. even though the role it plays as an inhibitor of catalytic activity is still under debate [15].

### 3.7. X-ray Diffraction (XRD) Analysis of the Decomposition Products

**Figure 5:**
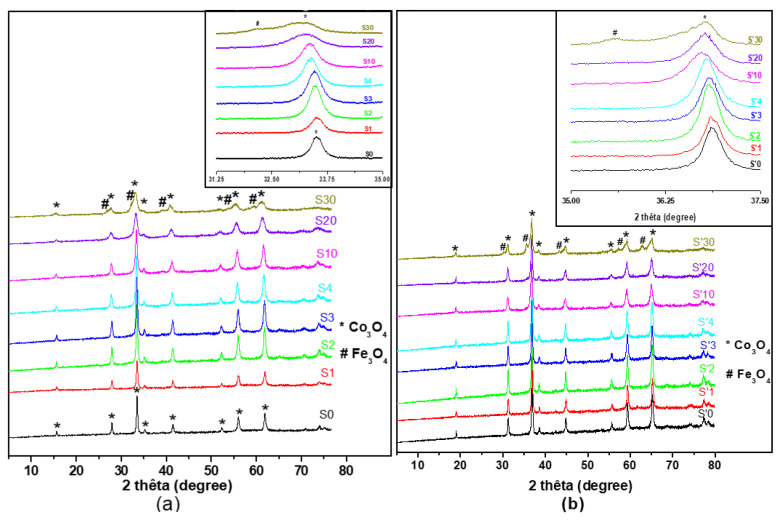
X-ray diffraction (XRD) of samples (**a**) S0 to S30, inset is zoom on the S samples highest peak; (**b**) S’0 to S’30 decomposition products, inset is zoom on the S’ samples highest peak. * Co_3_O_4_ diffraction peaks; # Fe_3_O_4_ diffraction peaks.

Figure 5 represents the XRD patterns of the thermally decomposed samples. All the diffraction peaks of sample S0 (S’0) have been perfectly indexed into the face centered cubic Co_3_O_4_ structures (space group Fd3m), without any trace of other phases. This observation corroborates IR spectra. For S samples S0 to S20 and S’ samples S’0 to S’10, there is no appearance of a new phase. A new phase, identified as Fe_3_O_4_ appears for S samples in S30 and S’ samples in S’20 (very small amount), and S’30.

The absence of a new phase for S samples, from S0 to S20 and for S’ samples, from S’0 to S’10, suggests the complete substitution of cobalt ions by iron ions in the Co_3_O_4_ crystalline structure. This is confirmed by a perceptible shift toward the lower 2θ values of the peaks of the XRD diffraction patterns of these materials (see the insets of Figure 5) in agreement with similar works [48]. These shifts correspond to the increase in the unit cell volume due to the substitution of cobalt by iron in the Co_3_O_4_ unit cell because the iron ion radius is slight larger than the cobalt ion radius (r(Fe^3+^) = 0.64 Å > r(Co^3+^) = 0.63 Å) for octahedral coordination [49].

**Figure 6:**
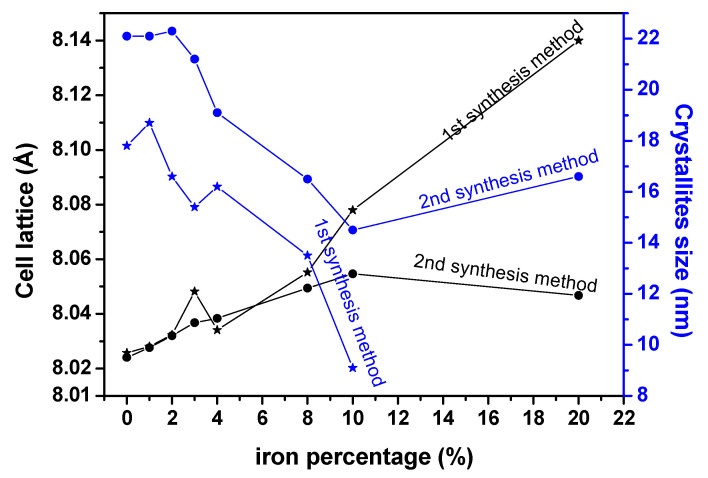
Variation of parameter lattice and crystallites size with iron percentage.

Figure 6 confirms the increase of the unit cell parameter of Fe^3+^-doped materials with the increasing iron percentage. It is also observed on Figure 5 that the width of the peaks increases with the quantity of Fe^3+^ ions for these doped materials. This indicates that the particle size is reduced for these doped materials. The size *D* of the crystallites was calculated by the Debye–Scherer equation with the most intense diffraction peak of the samples.

(1) $$D=\frac{\lambda }{\beta cos\theta \;}$$

where *λ* is the X-ray wavelength of the radiation (1.54 Å), *θ* is the diffraction angle of Bragg and *β* is the full width of the most intense peak.

Figure 6 shows an overall decrease of the crystallite size till the limit of substitution of iron inside the cobalt oxide crystalline structure. For S sample S30 and S’ samples S’20 and S’30, where a new phase has appeared, the increase of the substitution of iron inside Co_3_O_4_ yields to a distortion of diffraction patterns in such a way that determination of crystallites size or cell lattice parameters was not possible. However, for the first synthesis route, even beyond the limit of substitution of iron inside the cobalt oxide structure (S20), it seems that the crystallite size continues to decrease, as we can observe on the diffraction patterns which become broader. For both methods, the particle size values of these doped samples are around or less than 20 nm indicating that nanomaterial are obtained by both synthetic routes. According to the literature, the degree of substitution of a metal inside a crystalline structure of another metal oxide depends on the nature of those metals and the synthesis method. S. Angelov and al. synthetized Cu_x_Co_3−x_O_4_ by calcining the corresponding metals nitrates, and showed that beyond x = 0.9 (30% of metallic percentage), a new phase of CuO appears [50]. Li Gengnan et al. synthesized Fe_x_Co_3−x_O_4_ by co-precipitation method using oxalate as precipitant agent, and showed that the materials could be obtained for x = 1/3; 3/5; 1 (11,11; 20 and 33,33% of metallic percentage) [15]. Kwang Joo Kim et al. synthesized Fe_x_Co_3−x_O_4_ by spin coating mixed cobalt iron acetate dissolved in a solution 2-methoxyethanol as precursors and showed that the materials could be obtained for x = 0–2 [51]. Lontio et al. synthesized Zn_x_Ni_1−x_O by co-precipitation using malonate as precipitant agent and showed that beyond x = 0.15, a new phase ZnO appeared [27].

### 3.8. Raman Analysis of the Thermal Decomposition Products

**Figure 7:**
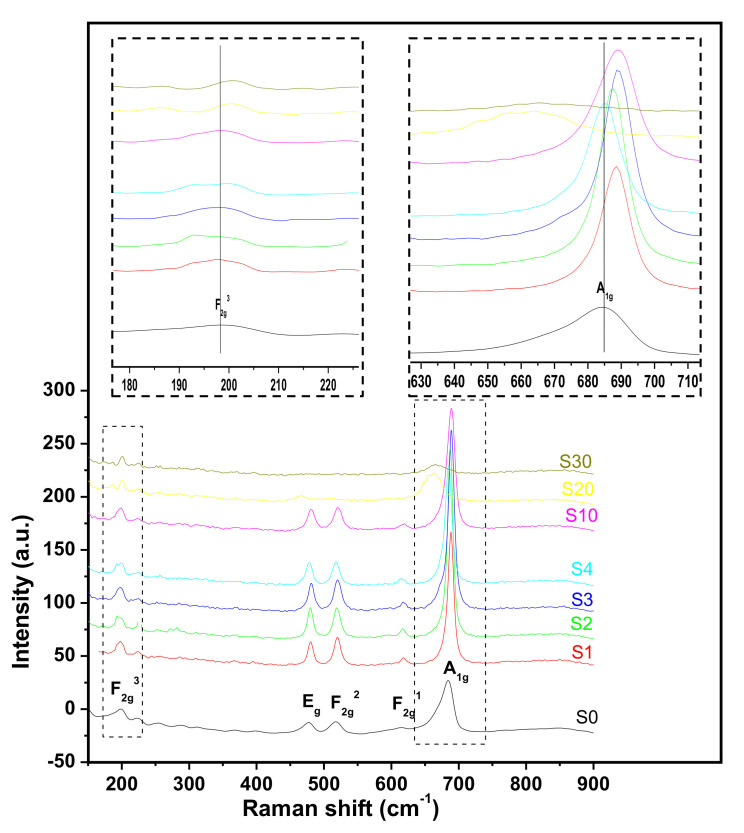
Raman spectra of samples S0, S1, S2, S3, S4, S10, S20 and S30 obtained by the first synthesis route. Inset is zoom of F_2g_^3^ and A_1g_ peaks located respectively at 200 cm^−1^ and 685.7 cm^−1^.

Raman spectroscopy is well known to be very sensitive to the microstructure of nanocrystal materials. Figure 7 gives the Raman spectrum of the decomposition products for samples synthesized by the first synthesis route. In accordance with the group theory prediction and other works reported on Co_3_O_4_ nanoparticles characterization [26,52,53,54], five main peaks are observed, 3F_2g_, E_g_, and A_1g_. The intense band at 685.7 cm^−1^ is characteristic of the symmetric stretching vibration of octahedral sites (CoO_6_) associated to the A_1g_ symmetry. The Raman bands with medium intensity located at 477.7 and 519.8 cm^−1^ indicate E_g_ and F_2g_^2^, respectively, while the weak band located at 613.4 cm^−1^ has F_2g_^1^ symmetry. The band at 199.7 cm^−1^ is attributed to the characteristics of the tetrahedral sites (CoO_4_) which are attributed to the F_2g_^3^ symmetry [52].

The random variation of the A_1g_ band position with increasing amount of iron percentage while F_2g_^3^ band position doesn’t shift suggests that the substitution of iron by cobalt take place predominantly in octahedral sites. Lattice disorder and low dimension crystal lead to the asymmetrical broadening and downshifting of A_1g_. However for samples S20 and S30, all the peaks become wider and are shifted towards a small wavenumber. This could be attributed to the particle size because of small particles scattering at so large angle that it becomes difficult to get defined peaks [53]. However, this analysis gives no new information about the obtained products (like the substitution of metal in a preferential metal site). It has not been performed on products obtained by the second synthesis route.

### 3.9. X-ray Photoelectron Spectroscopy (XPS) Analysis of the Thermal Decomposition Products

**Figure 8:**
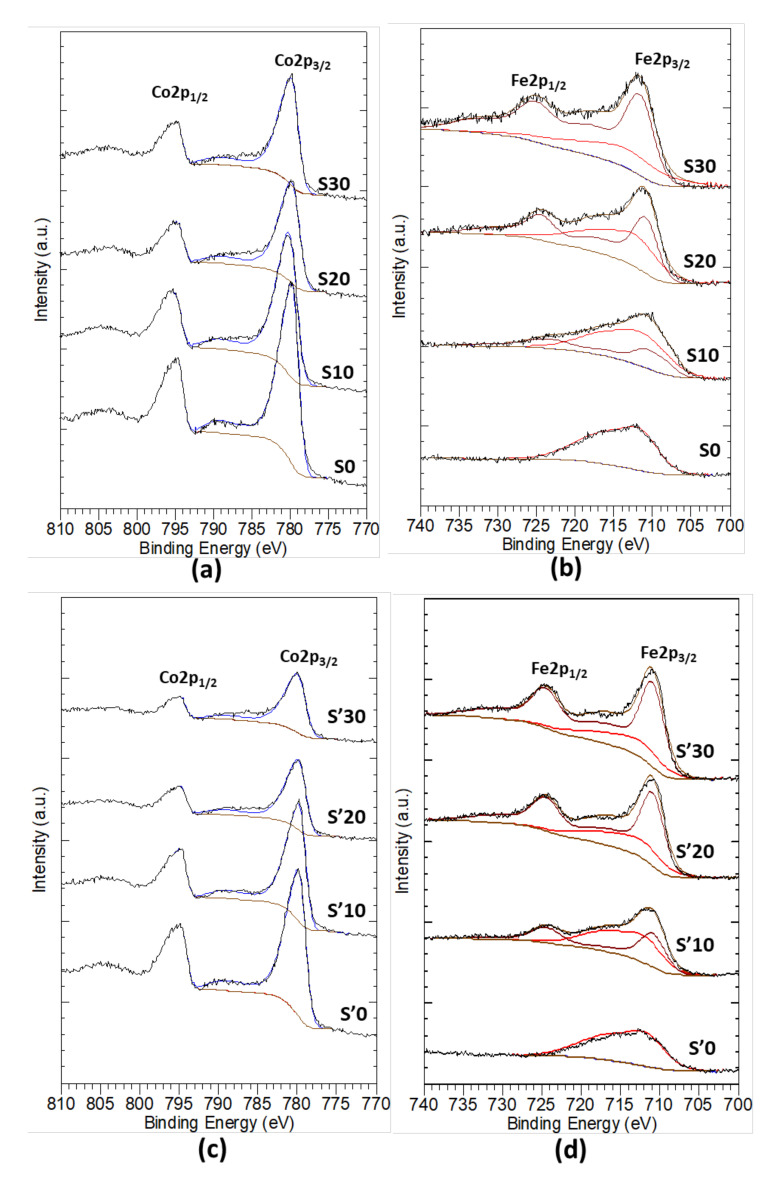
High-resolution X-ray photoelectron spectroscopy (XPS) spectra showing the Co 2p and Fe 2p lines of samples S0, S10, S20, S30 (**a**,**b**) and S’0, S’10, S’20, S’30 (**c**,**d**).

S Samples S0, S10, S20, S30 and S’ samples S’0, S’10, S’20, S’30 have been analyzed by XPS. Multiplet decomposition, described by Biesinger et al. [55], was used to fit the Fe 2p and Co 2p regions. Due to the complexity of this decomposition, only the global envelops corresponding to Co_3_O_4_ (blue) and Fe_3_O_4_ (brown) are shown here. Figure 8 displays the high-resolution spectrum corresponding to Co 2p and Fe 2p photoemissions. They show spin-orbit splitting into 2p1/2 and 2p3/2. Co 2p3/2 and Co 2p1/2 are located respectively at 780 and at 794.4 eV while Fe 2p3/2 and Fe 2p1/2 are located respectively at 711 and 724 eV. They result from the overlapping of the contribution of Co^2+^/Co^3+^ and Fe^2+^/Fe^3+^. Both 2p1/2 and 2p3/2 lines contain qualitatively the same chemical information [56]. Therefore, only the higher intensity lines with their shake-up satellite, for each element, are used to determine the contribution of each oxidation state.

Information about the relative proportion of each oxidation state of cobalt ion is usually determined by considering their satellite. The Cobalt(II) oxide has a strong shake-up satellite at about 5.9 eV (785 eV) above the Co 2p3/2 main peak which is absent in Co(III) complexes [57,58,59] or very low [56]. As Li Gengnan noted, for samples S0, S10, S20, S30 and samples S’0, S’10, S’20, S’30 we observe a progressive decrease of the contribution of those satellites with the increasing amount of iron, this implies an increasing of Co^3+^ species contribution at the surface of material [15]. It could be an important result since it has been proven that only octahedral sites occupied by Co^3+^ are responsible of the properties attributed to Co_3_O_4_ [12,60,61]. However, the previous analysis does not take into account that the insertion of a hetero atom modifies the environment around the targeted atom.

The binding energy of iron electrons emitted by the photoelectric effect overlaps with the one of cobalt electrons emitted by the Auger effect. Therefore, the XPS spectra of iron have been obtained by subtracting the contribution of Auger electrons of cobalt, fitted to the spectra of samples S0 and S’0, from all the Fe 2p spectra. One can observe the Fe 2p3/2 and Fe 2p1/2 lines at 711 and 725 eV, respectively, which are characteristic of iron(III) oxide [62,63]. The absence of peak at 707 eV indicates that there is no metallic iron on the surface. We note the absence of shake-up satellite at about 714 eV, which would be characteristic of iron (II) oxide. This suggests that almost all the iron atoms at the surface are in the Fe(III) state. However, according to the fact that this analysis considers that the contribution of cobalt Auger electrons in mixed sample S10, S20, S30 remains the same as in the pure sample S0, reservations have to be made.

The XPS spectra of O 1s are presented on Figure S1 (see supplementary information). The shape of the O 1s peaks shows the presence of at least two components. The first, which is relatively narrow and centered around 530.0 eV, is attributed to the lattice oxygen of the mixed cobalt-iron oxide phases. The second is much larger and centered around 532 eV. It can be assigned to the oxygen bound to contaminated carbon and to hydroxyl groups attached to the material surface [55]. This result corroborates well the FTIR analysis where the hydroxyl groups appear on the spectra.

Table 4 compares the expected values with the ICP and XPS data. The ICP analyses of samples obtained by the second synthesis route were performed on calcined samples.

According to the data presented in Table 4, the composition of the surface and the bulk are similar for samples obtained by the first synthesis route, except sample S30. It is explained by the appearance of the new phase Fe_3_O_4_. For the samples obtained by the second synthesis route, the Fe/Co ratio is higher at the surface than in the bulk. This reveals a strong tendency of iron to migrate at the surface of the material for sample S’10 (obtained: 0.18, expected: 0.11), where iron is completely inserted inside the cobalt oxide structure. The difference between expected and obtained Fe/Co ratio is even more important for sample S’20 (obtained: 0.64, expected: 0.25) and S’30 (obtained: 0.84; expected: 0.43). Again, this can tentatively be attributed to the formation of the separate Fe_3_O_4_ phase.

### 3.10. ToF-SIMS Analysis of the Thermal Decomposition Products

**Figure 9:**
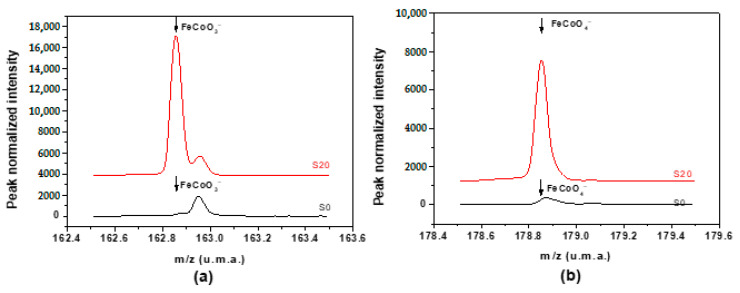
Partial negative spectra of S0 and S20 decomposition products showing the (**a**) FeCoO_3_^−^; (**b**) FeCoO_4_^−^ peak. The peak’s intensities are normalized with the respect to the total spectrum intensity (total spectrum).

The presence of iron in the Co_3_O_4_ spinel structure is corroborated by ToF-SIMS. Figure 9 shows the partial negative secondary ion mass spectra corresponding to the mass ranges of ions FeCoO_3_^−^ and FeCoO_4_^−^, for samples S0 and S20 after calcination. The identification of these two mixed-metal ion peaks, absent from the pure cobalt oxide sample S0, indicates the proximity of the two types of atoms at the nanoscale in sample S20.

Indeed, in SIMS, 10–30 Bi_3−5_^+^ primary ions eject matter from a hemispherical crater that is smaller than 10 nm of radius in inorganic materials such as metals or their oxides (e.g., 4–5 nm for 10 KeV Bi_3−5_ impinging on Au [64]). The presence of those additional ion peaks, which contain both iron and cobalt, confirm that the two metals are present in close vicinity, most probably in the same ≥10 nm particles.

**Figure 10:**
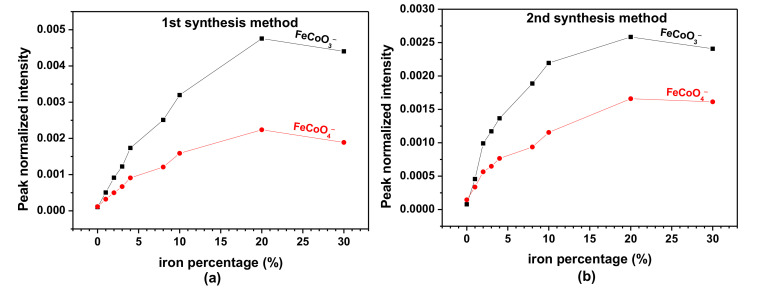
Variation of the intensities of the FeCoO_3_^−^ and FeCoO_4_^−^ ions normalized by the total spectrum intensity as a function of the Fe percentage for (**a**) the first synthetic route; (**b**) the second synthetic route.

The variation of the FeCoO_3_^−^ and FeCoO_4_^−^ peak intensities, normalized by the total spectrum intensities, with an increasing amount of iron was investigated and the results are presented in Figure 10.

For both synthetic routes, the intensities of the FeCoO_3_^−^ and FeCoO_4_^−^ ions increase with the ion percentage up to 20% of iron. The increase can be explained by the fact that, up to that point, all the iron (or almost all in the case of sample S’20) is inserted in the Co_3_O_4_ structure. This corroborates the XRD results, which indicate that there is only one crystalline phase of the spinel type up to 20% of substitution of cobalt by iron. However, for sample S30 and S’30, we observe a decrease of the normalized intensities of FeCoO_3_^−^ and FeCoO_4_^−^. This is explained by the fact that beyond 20% of insertion of iron inside Co_3_O_4_, the new phase Fe_3_O_4_ starts to occupy the analyzed surface. As the represented intensities are normalized by the total intensity, the decrease of FeCoO_3_^−^ and FeCoO_4_^−^ intensity simply mirrors the gradual reduction of the surface area covered by the mixed oxide in favor of the Fe_3_O_4_ phase. Lontio Fomekong et al. have reported similar observations when studying the insertion of Zn inside the NiO structure [27].

The presence of an excess of iron, at the surface of the decomposition products, obtained by the second synthesis method was also corroborated by ToF-SIMS results. Figure S2 (confer supporting information) compares the intensity ratios of $\frac{FeO_{2}^{-}}{FeO_{2}^{-}+CoO_{2}^{-}}$ with increasing amount of iron, obtained by the two synthesis methods. The intensity ratios of $\frac{FeO_{2}^{-}}{FeO_{2}^{-}+CoO_{2}^{-}}$ increases with the amounts of iron, for both S and S’ samples. However, it is obvious that this ratio is higher for S’ samples (S’10, S’20, S’30) than for S samples (S10, S20, S30).

### 3.11. Transmission Electron Microscopy (TEM) Analysis of the Decomposition Products

**Figure 11:**
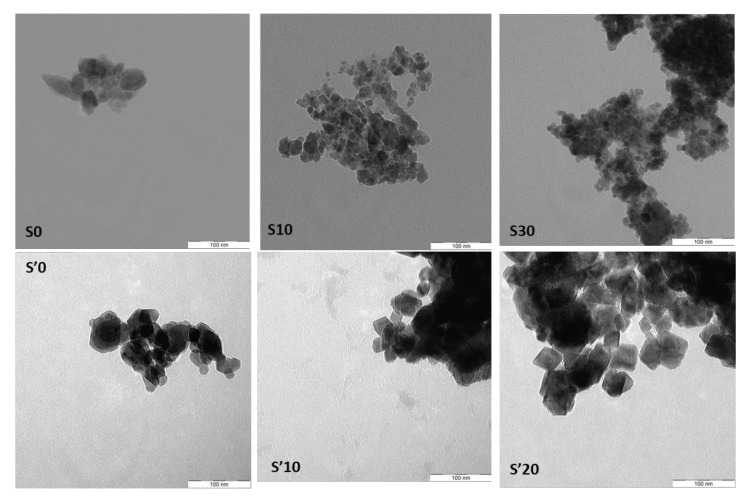
Transmission electron microscopy (TEM) images of the decomposition products S0, S10, S30 and S’0, S’10, S’20.

TEM images of the obtained samples (Figure 11) reveal a polycrystalline nature. For sample S0, S10 and S30 obtained with the first synthesis method, the TEM images show that there is not a well-defined and uniform shape. However, a tendency toward hexagonal shapes is observed. Sample S’0 and S’10 obtained by the second synthetic route exhibit a more uniform morphology. A closer look at the crystal edges also reveals hexagonal shapes for those two samples. In contrast, S’20 displays diamond shapes. This suggests that the crystallite shapes are better defined using the second synthesis route, with shapes varying with the amount of iron in the sample. The crystallite sizes revealed by TEM are in the sub-50 nm range, with some variation across the synthetic routes and iron percentages and a distribution of sizes in each sample. This roughly corroborates the estimates from XRD analysis which, for instance, provided values of 20 nm for samples S0 and S’0. In the literature, Co_3_O_4_ particles have been synthesized with various morphologies such as hollow nanospheres [65], nanowires [66], nanotubes [67,68] and octahedrons [69]. The properties of Co_3_O_4_ nanoparticles are known to vary with their shape, size and crystallization conditions.

## 4. Conclusions

Nanoparticles of mixed cobalt-iron oxide, (Co_(1−x)_Fe_x_)_3_O_4_ (x = 0–0.1), and the composite materials, (Co_(1−x)_Fe_x_)_3_O_4_/Fe_3_O_4_ (x > 0.2), have been obtained by green synthesis via co-precipitation at a relatively low temperature (400–600 °C) using, for the first time, a hetero-metal carboxylate precursor extracted from palm kernel oil, a Cameroonian local oil product. For the synthesis of the hetero-metallic carboxylate precursors from the oil, two methods were employed, the first in which the excess OH^−^ ions in solution were not neutralized and the second in which neutralization was carried out. The nature of both types of precursor was partially elucidated using Fourier transform infrared (FTIR) spectroscopy, thermogravimetric analysis (TGA), gas chromatography (GC) and ICP-AES while the structure and stoichiometry of the hetero-metal oxide nanoparticles, obtained from the precursors by thermal decomposition, were determined by XRD, FTIR, EDX-SEM, ToF-SIMS, XPS and TEM. XRD results, in particular, showed that the particle sizes of the calcination products (hetero-metal oxides) obtained from the neutralized precursors decreased from 22 to 14 nm with increasing substitution of Fe in the Co_3_O_4_ lattice while those from the non-neutralized precursors were agglomerated and decreased from 20 to 9 nm. XRD and Raman spectroscopy show that the particles obtained are crystalline while ToF-SIMS confirms the presence of Fe in the Co_3_O_4_ lattice. XPS and ToF-SIMS indicate that the composition in the bulk and the surface materials remains the same for samples obtained using the first synthesis method while for the second synthesis method, iron is more concentrated on the surface.

The availability of palm kernel oil in copious quantities all over Cameroon implies that these (hetero-metal oxides) and other such materials could be synthesized on an industrial scale.

## Figures and Tables

**Table 1:** Amounts of the metallic reagents used during the synthesis.

Reagents (g)	S0	S1	S2	S3	S4	S10	S20	S30
CoCl_2_.6H_2_O	0.602	0.593	0.584	0.576	0.567	0.516	0.438	0.367
Fe(NO_3_)_3_·9H_2_O	0	0.010	0.020	0.030	0.040	0.097	0.186	0.267
	S’0	S’1	S’2	S’3	S’4	S’10	S’20	S’30
Co(NO_3_)_2_·6H_2_O	0.243	0.241	0.238	0.236	0.233	0.219	0.194	0.170
Fe(NO_3_)_3_.9H_2_O	0.000	0.003	0.007	0.010	0.013	0.034	0.067	0.101

**Table 2:** Comparison between expected and obtained mole ratio of metal in the precursors.

		Elements	S0/S’0	S1/S’1	S2/S’2	S3/S’3	S4/S’4	S10/S’10	S20/S’20	S30/S’30
Found experimentally	First synthesis route	Fe/Co	0.000	0.0103	0.0210	0.0319	0.0428	0.113	0.247	0.414
	Second synthesis route	Fe/Co	0.000	0.0116	0.0200	0.0315	0.0396	0.0974	0.219	0.440
calculated		Fe/Co	0.000	0.0101	0.0204	0.0309	0.0417	0.111	0.250	0.429

**Table 3:** Carboxylate contents in the metallo-organic precursors and the palm kernel oil.

Acids	Palm Kernel Oil (%)	Metallo-Organic Precursors (%)
Lauric acid, C12:0	47.16	52.75
Myristic acid, C14:0	16.89	18.60
Oleic acid, C18:1; cis9	13.90	11.05
Palmitic acid, C16:0	8.48	9.35
Caprilic acid, C8:0	4.11	1.34
Capric acid, C10:0	3.59	2.85
Linoleic acid, C18:2; C9C12	2.29	0.22
Stearic acid, C18:0	2.79	2.91
Caproic acid, C6:0	0.36	0.26
Arachidic acid, C20:0	0.12	0.12
Oleic acid C18:1; cis11	0.11	0.09
Tridecylic acid C13:0	0.10	0.12
Arachidic acid C20:1; C11	0.08	0.05
Pentadecylic acid C15:0	0.02	0.03
Oleic acid C18:1; trans9	0.00	0.10
Arachidic acid C20:2; C11, C14	0.00	0.10

**Table 4:** Comparison of results of atomic Fe/Co ratio obtained by different analysis techniques.

		1st Synthesis Method				2nd Synthesis Method			
	Elements	S0	S10	S20	S30	S’0	S’10	S’20	S’30
Calculated	Fe/Co	0	0.11	0.25	0.43	0	0.11	0.25	0.43
ICP results	Fe/Co	0	0.11	0.25	0.41	0	0.12	0.24	0.48
XPS results	Fe/Co	0	0.12	0.27	0.65	0	0.18	0.64	0.84

## Data Availability

Data is contained within the article or Supplementary Materials.

## Supplementary Materials

The following are available online at https://www.mdpi.com/article/10.3390/nano11112833/s1, Figure S1. High resolution XPS spectra showing the O 1s lines of samples (a) S0, S10, S20, S30 and (b) S′0, S′10, S′20, S′30. Figure S2. SIMS intensity ratio FeO_2_^−^/(FeO_2_^−^ + CoO_2_^−^) with the increasing amount of iron, measured on calcined samples obtained with the 1st synthesis method and the 2nd synthesis method.

## References

[1] Roduner E. (2006). Size matters: Why nanomaterials are different. Chem. Soc. Rev..

[2] Shahid R. (2012). Green Chemical Synthesis of II-VI Semiconductor Quantum Dots. Ph.D. Thesis.

[3] Sahoo P., Djieutedjeu H., Poudeu P.F.P. (2013). Co_3_O_4_ nanostructures: The effect of synthesis conditions on particles size, magnetism and transport properties. J. Mater. Chem. A.

[4] Lou X., Han J., Chu W., Wang X., Cheng Q. (2007). Synthesis and photocatalytic property of Co_3_O_4_ nanorods. Mater. Sci. Eng. B.

[5] Maniak G., Stelmachowski P., Stanek J.J., Kotarba A., Sojka Z. (2011). Catalytic properties in N_2_O decomposition of mixed cobalt–iron spinels. Catal. Commun..

[6] Yamaura H., Tamaki J., Moriya K., Miura N., Yamazoe N. (1997). Highly Selective CO Sensor Using Indium Oxide Doubly Promoted by Cobalt Oxide and Gold. J. Electrochem. Soc..

[7] Chidambaram K., Malhotra L.K., Chopra K.L. (1982). Spray-pyrolysed cobalt black as a high temperature selective absorber. Thin Solid Films.

[8] Kim H.-K., Seong T.-Y., Lim J.-H., Ii Cho W., Soo Yoon Y. (2001). Electrochemical and structural properties of radio frequency sputtered cobalt oxide electrodes for thin-film supercapacitors. J. Power Sources.

[9] Makhlouf S.A. (2002). Magnetic properties of Co_3_O_4_ nanoparticles. J. Magn. Magn. Mater..

[10] Maruyama T., Arai S. (1996). Electrochromic Properties of Cobalt Oxide Thin Films Prepared by Chemical Vapor Deposition. J. Electrochem. Soc..

[11] Salavati-Niasari M., Mir N., Davar F. (2009). Synthesis and characterization of Co_3_O_4_ nanorods by thermal decomposition of cobalt oxalate. J. Phys. Chem. Solids.

[12] Omata K., Takada T., Kasahara S., Yamada M. (1996). Active site of substituted cobalt spinel oxide for selective oxidation of COH_2_. Part II. Appl. Catal. A Gen..

[13] Abu-Zied B.M., Bawaked S.M., Kosa S.A., Schwieger W. (2015). Effect of Pr, Sm, and Tb Doping on the Morphology, Crystallite Size, and N_2_O Decomposition Activity of Co_3_O_4_ Nanorods. J. Nanomater..

[14] Yan L., Ren T., Wang X., Ji D., Suo J. (2003). Catalytic decomposition of N_2_O over M_x_Co_1−x_Co_2_O_4_ (M = Ni, Mg) spinel oxides. Appl. Catal. B Environ..

[15] Li G., Li L., Li Y., Shi J. (2014). Highly Moisture-resistant Fe-doped Mesoporous Co_3_O_4_ Catalyst for Efficient Low-temperature CO Oxidation. New J. Chem..

[16] Manickam M., Ponnuswamy V., Sankar C., Suresh R., Mariappan R., Chandrasekaran J. (2017). Structural, optical, electrical and electrochemical properties of Fe: Co_3_O_4_ thin films for supercapacitor applications. J. Mater. Sci. Mater. Electron..

[17] Stella C., Soundararajan N., Ramachandran K. (2015). Structural, optical, and magnetic properties of Mn and Fe-doped Co_3_O_4_ nanoparticles. AIP Adv..

[18] Li G., Chen M., Ouyang Y., Yao D., Lu L., Wang L., Xia X., Lei W., Chen S.-M., Mandler D. (2019). Manganese doped Co_3_O_4_ mesoporous nanoneedle array for long cycle-stable supercapacitors. Appl. Surf. Sci..

[19] Deng S., Xiao X., Chen G., Wang L., Wang Y. (2016). Cd doped porous Co_3_O_4_ nanosheets as electrode material for high performance supercapacitor application. Electrochim. Acta.

[20] UmaSudharshini A., Bououdina M., Venkateshwarlu M., Dhamodharan P., Manoharan C. (2021). Solvothermal synthesis of Cu-doped Co_3_O_4_ nanosheets at low reaction temperature for potential supercapacitor applications. Appl. Phys. A.

[21] Hao J., Peng S., Li H., Dang S., Qin T., Wen Y., Huang J., Ma F., Gao D. (2018). A low crystallinity oxygen-vacancy-rich Co_3_O_4_ cathode for high-performance flexible asymmetric supercapacitors. J. Mater. Chem. A.

[22] Xiong S., Weng S., Tang Y., Qian L., Xu Y., Li X., Lin H., Xu Y., Jiao Y., Chen J. (2021). Mo-doped Co_3_O_4_ ultrathin nanosheet arrays anchored on nickel foam as a bi-functional electrode for supercapacitor and overall water splitting. J. Colloid Interface Sci..

[23] Niu M., Wang Y., Cheng Y., Chen G., Cui L. (2009). Fabrication of Co_3_O_4_ cubic nanoframes: Facet-preferential chemical etching of Fe^3+^ ions to Co_3_O_4_ nanocubes. Mater. Lett..

[24] Thota S., Kumar A., Kumar J. (2009). Optical, electrical and magnetic properties of Co_3_O_4_ nanocrystallites obtained by thermal decomposition of sol–gel derived oxalates. Mater. Sci. Eng. B.

[25] Yang Y.-P., Liu R.-S., Huang K.-L., Wang L.-P., Liu S.-Q., Zeng W.-W. (2007). Preparation and electrochemical performance of nanosized Co_3_O_4_ via hydrothermal method. Trans. Nonferrous Met. Soc. China.

[26] Gu F., Li C., Hu Y., Zhang L. (2007). Synthesis and optical characterization of Co_3_O_4_ nanocrystals. J. Cryst. Growth.

[27] Fomekong R.L., Tsobnang P.K., Magnin D., Hermans S., Delcorte A., Ngolui J.L. (2015). Coprecipitation of nickel zinc malonate: A facile and reproducible synthesis route for Ni_1− x_Zn_x_O nanoparticles and Ni_1− x_Zn_x_O/ZnO nanocomposites via pyrolysis. J. Solid State Chem..

[28] Ansari F., Soofivand F., Salavati-Niasari M. (2015). Utilizing maleic acid as a novel fuel for synthesis of PbFe_12_O_19_ nanoceramics via sol–gel auto-combustion route. Mater. Charact..

[29] Das S., Srivastava V. (2016). Synthesis and characterization of copper succinate and copper oxide nanoparticles by electrochemical treatment: Optimization by Taguchi robust analysis. Can J. Chem. Eng..

[30] Sun S., Zeng H. (2002). Size-controlled synthesis of magnetite nanoparticles. J. Am. Chem. Soc..

[31] Jović Orsini N., Babić-Stojić B., Spasojević V., Calatayud M.P., Cvjetićanin N., Goya G.F. (2018). Magnetic and power absorption measurements on iron oxide nanoparticles synthesized by thermal decomposition of Fe(acac)_3_. J. Magn. Magn. Mater..

[32] Kamta H., Kenfack T.P., Lontio F.R., Etape E., Joy P., Delcorte A., Lambi J. (2018). Structural characterization and magnetic properties of undoped and copper-doped cobalt ferrite nanoparticles prepared by the octanoate coprecipitation route at very low dopant concentrations. RSC Adv..

[33] Borel N.N.M., Foba-Tendo J., Yufanyi D.M., Etape E.P., Eko J.N., Ngolui L.J. (2014). Averrhoa carambola: A renewable source of oxalic acid for the facile and green synthesis of divalent metal (Fe, Co, Ni, Zn, and Cu) oxalates and oxide nanoparticles. J. Appl. Chem..

[34] Sharma J.K., Srivastava P., Singh G., Akhtar M.S., Ameen S. (2015). Green synthesis of Co_3_O_4_ nanoparticles and their applications in thermal decomposition of ammonium perchlorate and dye-sensitized solar cells. Mater. Sci. Eng. B.

[35] Vani P., Manikandan N., Vinitha G. (2017). A green strategy to synthesize environment friendly metal oxide nanoparticles for potential applications: A review. Asian J. Pharm. Clin. Res..

[36] Das R.K., Golder A.K. (2017). Co_3_O_4_ spinel nanoparticles decorated graphite electrode: Bio-mediated synthesis and electrochemical H_2_O_2_ sensing. Electrochim. Acta.

[37] Saeed M., Akram N., Naqvi S.A.R., Usman M., Abbas M.A., Adeel M., Nisar A. (2019). Green and eco-friendly synthesis of Co_3_O_4_ and Ag-Co_3_O_4_: Characterization and photo-catalytic activity. Green Process. Synth..

[38] Kombaiah K., Vijaya J.J., Kennedy L.J., Kaviyarasu K., Ramalingam R.J., Al-Lohedan H.A. (2019). Green synthesis of Co_3_O_4_ nanorods for highly efficient catalytic, photocatalytic, and antibacterial activities. Int. J. Nanosci. Nanotechnol..

[39] Waris A., Din M., Ali A., Afridi S., Baset A., Khan A.U., Ali M. (2021). Green fabrication of Co and Co_3_O_4_ nanoparticles and their biomedical applications: A review. Open Life Sci..

[40] Rasheed T., Nabeel F., Bilal M., Iqbal H. (2019). Biogenic synthesis and characterization of cobalt oxide nanoparticles for catalytic reduction of direct yellow-142 and methyl orange dyes. Biocatal. Agric. Biotechnol..

[41] Hsu C.-M., Huang Y.-H., Chen H.-J., Lee W.-C., Chiu H.-W., Maity J.P., Chen C.-C., Kuo Y.-H., Chen C.-Y. (2018). Green synthesis of nano-Co_3_O_4_ by Microbial Induced Precipitation (MIP) process using Bacillus pasteurii and its application as supercapacitor. Mater. Today Commun..

[42] Koyyati R., Kudle K.R., Padigya P.R.M. (2016). Evaluation of antibacterial and cytotoxic activity of green synthesized cobalt nanoparticles using *Raphanus sativus* var. longipinnatus leaf extract. Int. J. Pharmtech Res..

[43] Berger K.G., Caballero B. (2003). Palm kernel oil. Encyclopedia of Food Sciences and Nutrition.

[44] Kapseu C. (2009). Production, analyse et applications des huiles végétales en Afrique. Oléagineux Corps Gras Lipides.

[45] Pantzaris T., Ahmad M.J. (2001). Properties and utilization of palm kernel oil. Palm Oil Dev..

[46] Folch J., Lees M., Stanley G.S. (1957). A simple method for the isolation and purification of total lipides from animal tissues. J. Biol. Chem..

[47] Hassim N.A.M., Dian L. (2017). Usage of palm oil, palm kernel oil and their fractions as confectionery fats. J. Oil Palm Res..

[48] Li G.-H., Dai L.-Z., Lu D.-S., Peng S.-Y. (1990). Characterization of copper cobalt mixed oxide. J. Solid State Chem..

[49] Pan K.-L., Overstreet W.C., Robinson K., Hubert A.E., Crenshaw G.L. (1980). Equivalent Uranium and Selected Minor Elements in Magnetic Concentrates from the Candle Quadrangle, Solomon Quadrangle, and Elsewhere in Alaska. https://pascal-francis.inist.fr/vibad/index.php?action=getRecordDetail&idt=PASCALGEODEBRGM8220230354.

[50] Angelov S., Zhecheva E., Petrov K., Menandjiev D. (1982). The properties of a spinel copper cobaltite prepared at low temperatures and normal pressure. Mater. Res. Bull..

[51] Kim K.J., Kim H.K., Park Y.R., Ahn G.Y., Kim C.S., Park J.Y. (2006). Magnetic and optical properties of spinel Fe_x_Co_3−x_O_4_ thin films. J. Magn. Magn. Mater..

[52] Gawali S.R., Gandhi A.C., Gaikwad S.S., Pant J., Chan T.S., Cheng C.L., Ma Y.R., Wu S.Y. (2018). Role of cobalt cations in short range antiferromagnetic Co_3_O_4_ nanoparticles: A thermal treatment approach to affecting phonon and magnetic properties. Sci. Rep..

[53] Rashad M., Rüsing M., Berth G., Lischka K., Pawlis A. (2013). CuO and Co_3_O_4_ Nanoparticles: Synthesis, Characterizations, and Raman Spectroscopy. J. Nanomater..

[54] Blakemore J.D., Gray H.B., Winkler J.R., Müller A.M. (2013). Co_3_O_4_ Nanoparticle Water-Oxidation Catalysts Made by Pulsed-Laser Ablation in Liquids. ACS Catal..

[55] Biesinger M.C., Payne B.P., Grosvenor A.P., Lau L.W.M., Gerson A.R., Smart R.S.C. (2011). Resolving surface chemical states in XPS analysis of first row transition metals, oxides and hydroxides: Cr, Mn, Fe, Co and Ni. Appl. Surf. Sci..

[56] Artyushkova K., Levendosky S., Atanassov P., Fulghum J. (2007). XPS Structural Studies of Nano-composite Non-platinum Electrocatalysts for Polymer Electrolyte Fuel Cells. Top. Catal..

[57] Yang J., Liu H., Martens W.N., Frost R.L. (2010). Synthesis and characterization of cobalt hydroxide, cobalt oxyhydroxide, and cobalt oxide nanodiscs. J. Phys. Chem. C.

[58] McIntyre N.S., Cook M.G. (1975). X-ray photoelectron studies on some oxides and hydroxides of cobalt, nickel, and copper. Anal. Chem..

[59] Casella I.G., Guascito M.R. (1999). Anodic electrodeposition of conducting cobalt oxyhydroxide films on a gold surface. XPS study and electrochemical behaviour in neutral and alkaline solution. J. Electroanal. Chem..

[60] Jacobs J.P., Maltha A., Reintjes J.G.H., Drimal J., Ponec V., Brongersma H.H. (1994). The surface of catalytically active spinels. J. Catal..

[61] Shelef M., Wheeler M.A.Z., Yao H.C. (1975). Ion scattering spectra from spinel surfaces. Surf. Sci..

[62] Brundle C., Chuang T., Wandelt K. (1977). Core and valence level photoemission studies of iron oxide surfaces and the oxidation of iron. Surf. Sci..

[63] Graat P.C., Somers M.A. (1996). Simultaneous determination of composition and thickness of thin iron-oxide films from XPS Fe 2p spectra. Appl. Surf. Sci..

[64] Delcorte A., Leblanc C., Poleunis C., Hamraoui K. (2013). Computer Simulations of the Sputtering of Metallic, Organic, and Metal–Organic Surfaces with Bin and C60 Projectiles. J. Phys. Chem. C.

[65] Wang X., Zhong Y., Zhai T., Guo Y., Chen S., Ma Y., Yao J., Bando Y., Golberg D. (2011). Multishelled Co_3_O_4_-Fe_3_O_4_ hollow spheres with even magnetic phase distribution: Synthesis, magnetic properties and their application in water treatment. J. Mater. Chem..

[66] Xiao X., Liu X., Zhao H., Chen D., Liu F., Xiang J., Hu Z., Li Y. (2012). Facile shape control of Co_3_O_4_ and the effect of the crystal plane on electrochemical performance. Adv. Mater..

[67] Shen X.-P., Miao H.-J., Zhao H., Xu Z. (2008). Synthesis, characterization and magnetic properties of Co_3_O_4_ nanotubes. Appl. Phys. A.

[68] Wang R., Liu C., Zhang H., Chen C., Guo L., Xu H., Yang S. (2004). Porous nanotubes of Co_3_O_4_: Synthesis, characterization, and magnetic properties. Appl. Phys. Lett..

[69] Chen Y.H., Zhou J.F., Mullarkey D., O’Connell R., Schmitt W., Venkatesan M., Coey M., Zhang H.Z. (2015). Synthesis, characterization and magnetic properties of ultrafine Co_3_O_4_ octahedra. AIP Adv..

