# Supplementary material for: Green Synthesis of Iron-Doped Cobalt Oxide Nanoparticles from Palm Kernel Oil via Co-Precipitation and Structural Characterization
Source: Nanomaterials (Basel). 2021 Oct 25;11(11):2833. doi: 10.3390/nano11112833 (PMC8617965; doi:10.3390/nano11112833)
Supplement: Supplementary file 1 [file nanomaterials-11-02833-s001.zip › nanomaterials-1407953-supplementary.pdf]

nanomaterials

MDPI

Supplementary Materials

Green Synthesis of Iron-Doped Cobalt Oxide Nanoparticles from Palm Kernel Oil via Co-precipitation and Structural Characterization

Cedrik Ngnintedem Yonti [1,2] Patrice Kenfack Tsobnang [3], Roussin Lontio Fomekong [4*], Francois Devred [2], Eric Mignolet [5], Yvan Larondelle [5], Sophie Hermans [2], Arnaud Delcorte [2] and John Lambi Ngolui [4]

[1] Inorganic Chemistry Department, University of Yaoundé I, 812 Yaoundé, Cameroon; cedrik.ngnintedem@uclouvain.be
[2] Institute of Condensed Matter and Nanosciences, Catholic University of Louvain, Croix du Sud 1, 1348 Louvain-la-Neuve, Belgium; sophie.hermans@uclouvain.be (S.H.); arnaud.delcorte@uclouvain.be (A.D.); francois.devred@uclouvain.be (F.D.)
[3] Chemistry Department, Faculty of Science, University of Dschang, 67 Dschang, Cameroon; pakenfack@gmail.com
[4] Chemistry Department, Higher Teacher Training College, University of Yaoundé I, 47 Yaoundé, Cameroon; jngolui@gmail.com (J.L.N)
[5] Louvain Institute of Biomolecular Science and Technology, Catholic University of Louvain, Croix du Sud 4-5, 1348 Louvain-la-Neuve, Belgium; eric.mignolet@uclouvain.be (E.M.); yvan.larondelle@uclouvain.be (Y.L.)
* Correspondence: lonforou@yahoo.fr (R.L.F)

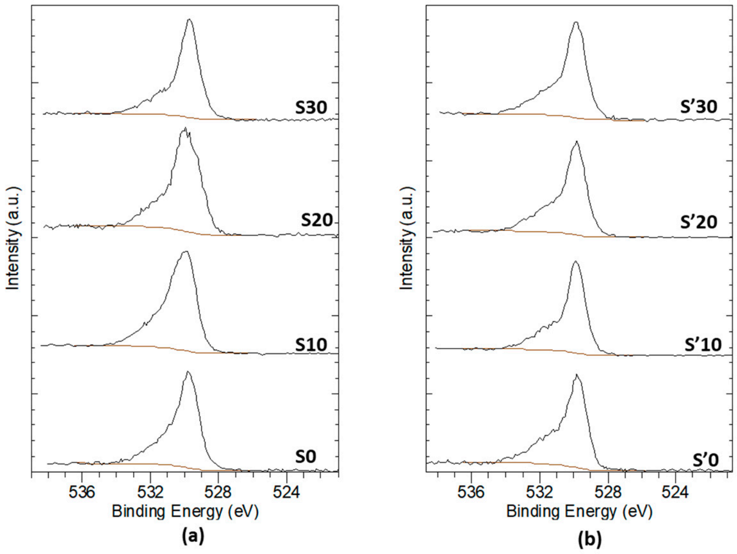

Figure S1. High resolution XPS spectra showing the O 1s lines of samples (**a**) S0, S10, S20, S30 and (**b**) S'0, S'10, S'20, S'30.

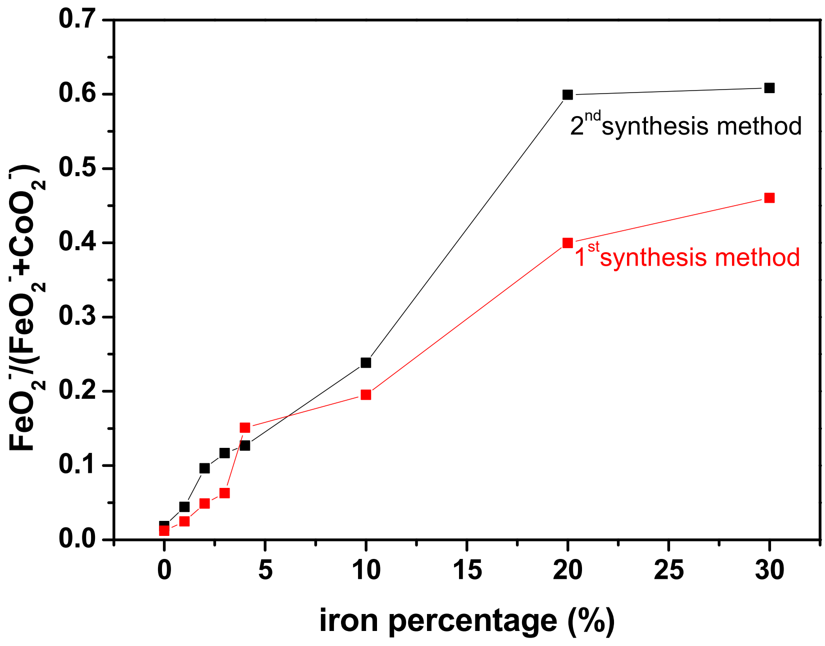

Figure S2. SIMS intensity ratio $FeO_2^-/(FeO_2^- + CoO_2^-)$ with the increasing amount of iron, measured on calcined samples obtained with the 1st synthesis method and the 2nd synthesis method.
